# Castration-Resistant Prostate Cancer: Mechanisms, Targets, and Treatment

**DOI:** 10.1155/2012/327253

**Published:** 2012-03-05

**Authors:** Teresa Maria Santos Amaral, Daniela Macedo, Isabel Fernandes, Luis Costa

**Affiliations:** Oncology Division, Hospital de Santa Maria, Instituto de Medicina Molecular, 1649-039 Lisbon, Portugal

## Abstract

Patients with castration-resistant prostate cancer (CRPC), who progress after docetaxel therapy, had until very recently, only a few therapeutic options. Recent advances in this field brought about new perspectives in the treatment of this disease. Molecular, basic, and translational research has given us a better understanding on the mechanisms of CRPC. This great investment has turned into a more rational approach to the development of new drugs. Some of the new treatments are already available to our patients outside clinical trials and may include inhibitors of androgen biosynthesis; new chemotherapy agents; bone-targeted therapy; and immunotherapy. This paper aims to review the mechanisms of prostate cancer resistance, possible therapeutic targets, as well as new options to treat CRPC.

## 1. Introduction

Prostate cancer is the most common malignancy in males in Western countries, representing the second leading cause of cancer death [1]. Advances in screening and diagnosis have allowed detection of the disease in early stages (approximately 85% of cases diagnosed), stages at which the therapeutic options are curative and include surgery, radiation and, in some cases, active surveillance only [2–4]. However, for late-stage disseminated disease, current therapies are merely palliative. In 1941, a study of Huggins and Hodges showed the close relationship of androgens with prostate tumor growth and androgen-deprivation therapy (castration) became the key treatment for these stages in monotherapy or in combination with other methods [2, 4, 5]. Initial responses to castration therapy are quite favorable, with a significant clinical regression and rapid biochemical responses, as assessed by decline in levels of serum marker, prostate-specific antigen (PSA) in 80–90% of patients with metastatic disease [2, 4, 6]. Despite a good initial response, remissions last on average 2-3 years, with eventual progression occurring despite castration [4, 5, 7]. In these cases prostate cancer will progress to a castration-insensitive phase of disease (Castration-Resistant Prostate Cancer—CRPC) which carries a worse prognosis and translates into a survival time of 16–18 months in average from the beginning of progression [2, 4–6]. Systemic therapies have also been an option in the management to these patients. However, chemotherapy is not well tolerated by all CRPC patients, who were often elderly men with limited bone marrow reserve and concurrent medical conditions [8]. In 2004 the result of two major phase 3 clinical trials established docetaxel as the first-line chemotherapy regimen in advanced stage disease [6].

Treatment of patients with CRPC remains a significant clinical challenge. This paper aims to address the mechanisms of resistance in the context of CRPC, as well as new therapeutic targets, and a brief discussion of current and future treatments.

## 2. Mechanisms and Targets in CRPC

The key for the development of new drugs and to optimize androgenic suppression in advanced stages of CRPC is the identification and characterization of molecular targets and mechanisms that lead to tumor growth. Disease progression involves the development of cellular adaptive pathways of survival in an androgen-depleted environment [3]. Experimental evidence assigns an important role to the continuous activation of the androgenic receptors (ARs) in tumor growth, as well as alternative independent routes [2]. In general, resistance mechanisms can be divided into 6 groups.

**(i) Increased Expression of Enzymes Involved in Steroidogenesis**

Studies have suggested that, in CRPC patients, even castrate serum levels of androgen are still sufficient for AR activation and able to maintain cancer cells survival. Indeed, the intratumoral levels of testosterone in CRPC patients are equal of those found in noncastrate patients [4]. The source of these androgens is thought to be derived from the synthesis of androgens directly in prostate cancer cells due to an upregulation of the enzymes and activation of the routes necessary for the synthesis of androgens such as testosterone and dihydrotestosterone [3, 5, 9]. Also bone metastases contain intact enzyme pathways for conversion of adrenal androgens to testosterone and dihydrotestosterone [4]. Montgomery and colleagues showed that there was marked reversal of the DHT : testosterone ratio in the metastatic tumor. These tumor cells express significantly lower levels of SRD5A2, which catalyses the conversion of testosterone to DHT, and higher levels of UGT2B15 and UGT2B17, which mediate the irreversible glucuronidation of DHT metabolites. Marked up regulation of CYP19A1, which mediates the aromatization of testosterone to estradiol, was also observed in the metastases samples [3–5, 9].

**(ii) Increased Expression of AR**

The overexpression of AR have been involved in the progression of prostate cancer [3]. The activated AR pathways observed in these CRPC patients has been postulated as a result of genetic phenomena that promotes increased sensitivity of AR. DNA amplifications are responsible for AR overexpression and for its activation in presence of low levels of ligand (androgens) [3, 9].

**(iii) AR Gene Mutations and Altered Ligand Specificity**

While the androgens are the main factors of tumor growth and AR signaling, the presence of AR mutations leads to its activation by nonandrogenic steroid molecules and antiandrogens [3]. The majority AR mutations are point mutations in the AR ligand-binding domain, and initially this was considered relevant to explain why 10–30% of patients receiving antiandrogens treatment experience paradoxical PSA drop on cessation of treatment [5]. However the AR mutations could occur in other regions such as the amino terminus or the DNA binding domain that confer oncogenic properties to the AR [5]. At the present, the role of AR mutations in the anti-androgen withdrawal phenomena is called into questioned and a new explanation is offered since the discovery of alternative splicing of the AR. In fact, in recent reports [6, 7] it was shown that splice variants of AR with deletion of exons 5, 6, and 7 could result in AR capable to translocate to the nucleus without ligand binding.

**(iv) Downstream Signaling Receptor for Androgens**

One of the most important mechanisms in the development of castration resistance is the activation of different signal transduction pathways in CRPC cells. They could enhance the activity of the AR or its coactivators in the presence of low levels or even in the absence of androgen. These include other receptors such as epithelial growth factors, insulin growth factors, and tyrosine-kinase receptor [7].

**(v) Bypass Pathways**

The induction of bypass pathways independent of AR, is an important mechanism of castration resistance, that can overcame apoptosis induced by androgen-deprivation therapy. One such example of this is the up-regulation of antiapoptotic proteins, including the protein Bcl-2 gene [3, 10].

**(vi) Stem Cells**

Prostatic cancer stem cells are rare and undifferentiated cells that do not express AR on their surface, being independent of androgens to survive [3]. Currently it is thought that these cells can be responsible for maintaining tumor growth and development, because they are able to survive under androgen-deprivation therapy. The identification of these cells is possible based on the expression of surface protein (*α*1*β*1 integrin and CD133), which could allow new targets therapies [3].

## 3. Treatment Options

The growth of prostate cancer is originally androgen dependent and metastatic tumors are generally treated with androgen ablation therapy, with or without antiandrogen supplementation [2, 11, 12]. However, resistance to hormonal therapy occurs within 12–18 months (remissions last on average 2-3 years, progression occurs even under castration [4, 5, 9]), referred to as hormone-refractory or CRPC [2]. Resistance to hormones (in patients with metastatic disease) is probably shorter than 2-3 years, using PSA. In addition survival with CRPC is now longer than 16–18 months. Until recently, patients with castration-resistant prostate cancer had limited treatment options after docetaxel chemotherapy. However, in 2010, new options emerged [8]. The three nonhormonal systemic approaches that have been found to prolong survival are docetaxel as first line [13] chemotherapy, cabazitaxel as second-line cytotoxic chemotherapy [8, 14], and a vaccine named sipuleucel-T [15]. A new hormonal manipulation with abiraterone acetate [14] also showed to prolong survival in CRPC.

The current palliative treatment options for patients with CRPC can be divided in different groups such as secondary hormonal therapies, chemotherapy agents, vaccine-based immune therapy, bisphosphonates, radiotherapy and novel targets.

### 3.1. Hormonal Therapies

Drugs that reduce circulating levels of androgens or that competitively inhibit the action of androgens remain central to the treatment of prostate cancer. The surgical or medical castration with orchiectomy or gonadotropin-releasing hormone (GnRH) agonists, respectively, suppresses testicular testosterone generation. However, the duration of response to castration is short (12–33 months) and, in almost all patients, is followed by the emergence of a castration-resistant phenotype [3]. The combination with antiandrogens to achieve the maximum androgen blockade (MAB) did not prove to prolong survival and 30% of the patients have a drop in PSA after discontinuing antiandrogens [3, 16]. Maintenance of oral glucocorticoids at lower doses (10 mg/day) can result in temporary PSA responses for 25% of the patients, presumably due to adrenal androgen suppression [3, 17].

For patients whose disease progresses after a MAB, antiandrogen can be discontinued [18] or can be switched to an alternative antiandrogen as showed in several reports [18–20].

High-dose (150 mg daily) bicalutamide as second-line hormonal therapy resulted in ≥50% PSA reduction in 20%–45% of patients [18, 21–23].

Diethylstilboestrol (DES), a synthetic estrogen, as well as the other estrogens, suppresses the hypothalamic-pituitary-gonadal axis and it reduces ≥50% the total PSA in 26% to 66% of patients with CRPC. However, the thromboembolic toxicity limited is use [18, 24, 25].

Ketoconazol is an antifungal agent that can be given to CRPC patients after antiandrogen withdrawal because it inhibits cytochrome P-450 enzyme-mediated steroidogenesis in testes and adrenal glands and when given at high-dose (1200 mg/day) or low dose (600 mg/day) it resulted in ≥50% PSA reduction in 27% to 63% and 27 to 46%, of patients, respectively [18].

Abiraterone acetate, a prodrug of abiraterone, is potent and highly selective inhibitor of androgen biosynthesis that blocks cytochrome P450 c17 (CYP 17), a critical enzyme in testosterone synthesis, thereby blocking androgen synthesis by the adrenal glands and testes and within prostate tumor [26–30]. The Cou-AA-301 trial compared abiraterone acetate (1000 mg once daily) plus prednisone versus placebo plus prednisone in patients who had previously received docetaxel. This study randomly assigned 1195 patients and the results exceeded the preplanned criteria, with an overall survival longer in the abiraterone arm (14.8 months versus 10.9 months) (*P* < 0.0001) and with all secondary end points favoring the treatment group, including time to PSA progression (10.2 versus 6.6 months) (*P* < 0.001), progression-free survival (5.6 months versus 3.6 months) (*P* < 0.001), and PSA response rate (29% versus 6%) (*P* < 0.001) [26]. The adverse events more frequently related to abiraterone acetate than to placebo group were urinary tract infections, adverse events associated with elevated mineralocorticoid levels such as fluid retention and edema, hypokalemia, and hypertension, as well as cardiac disorders and liver-function test abnormalities [26].

MDV3100 is an androgen receptor antagonist which prevents nuclear translocation and recruitment of coactivators; it has been shown antitumor activity in men with CRPC after failure of prior hormonal therapy, in phase I/II trial [31, 32]. The AFFIRM trial (a phase III trial) compared MDV3100 versus placebo in patients with docetaxel-refractory CRPC. [18, 33–35]. A planned interim analysis of the AFFIRM trial revealed that estimated median survival was 18.4 months for men treated with MDV3100, compared with 13.6 months for men treated with placebo (*P* < 0.0001). This translates into a 37% reduction in the risk for death with MDV3100 (hazard ratio, 0.631). As a result, the trial's Independent Data Monitoring Committee recommended that AFFIRM should be stopped earlier and that men who were receiving placebo should be offered MDV3100. The recommendation was based on the fact that the study's prespecified interim efficacy stopping criteria were successfully met. The committee also examined the safety profile to date and determined that MDV3100 demonstrated a risk/benefit ratio that was favorable enough to stop the study.

The PREVAIL trial (A safety and efficacy study of oral MDV3100 in chemotherapy-naïve patients with progressive metastatic prostate cancer) is still ongoing and recruiting patients.

### 3.2. Bone-Targeted Therapy: Bisphosphonates and Denosumab

In men with advanced prostate cancer, the biphosphonate zoledronate has been shown to prevent or delay skeletal complications in men with bone metastases, as well as to palliate bone pain [36, 37]. At an average followup of 24 months, there was a significant reduction in the frequency of skeletal related events (SREs) in men receiving zoledronic acid compared to placebo (38 versus 49 percent), and the median time to develop an SRE was significantly longer with zoledronic acid (488 versus 321 days) [38]. Biphosphonates may also have a role in preventing osteopenia that frequently accompanies the use of androgen-deprivation therapy [39–41].

More recent data have show that denosumab is also an effective treatment for patients with CRPC and bone metastases. In a phase III study denosumab, a human monoclonal antibody against RANKL, was compared with zoledronic acid for prevention of skeletal-related events. The results showed advantage to denosumab, representing another treatment opportunity for CRPC patients [42].

### 3.3. External Beam Radiotherapy, Hemibody RT, and Radioisotope Pharmaceuticals

Focal external beam radiation therapy (RT) is a palliative treatment possibility that should be considered for men with CRPC and bone pain that is limited to one or a few sites. Several clinical trials as well as a systematic review of the literature suggest that single treatments with fractionation schedules provide palliation with cost effectiveness and patient convenience [43–46].

Hemibody RT could also be considered in selected patients with symptomatic disease limited to one side of the diaphragm, in order to rapid pain relief, when multiple bone metastases are present [47].

However, this technique has frequently been replaced by the administration of radioisotope pharmaceuticals which may be associated with less toxicity and are more appropriated for patients with multiple painful lesions [48].

In order for these patients to be treated with radioisotopes the presence of uptake on bone scan due to metastatic disease at sites that correlate with pain is necessary. These radioisotopes are used in men with advanced prostate cancer with osteoblastic bone metastasis. These patients are often characterized by a high ratio of bone to soft tissue metastases.

Multiple radioisotopes have been used but the most extensive data are with 89-strontium (89Sr), Radium-223 and 153-samarium (153Sm). Several clinical trials provide the rational for the use of this approach in carefully selected patients [49–55].

Radium-223 is an alpha-emitting pharmaceutical agent that showed to improve survival in a phase III study [56]. Compared with placebo, Radium-223 was associated with improved overall survival (median 14.0 versus 11.2 months; HR, 0.69; *P* = .002)

### 3.4. Chemotherapy

Docetaxel is the only approved chemotherapy that has been shown to prolong survival among men with metastatic CRPC. The trial TAX 327 compared chemotherapy with docetaxel plus prednisone versus mitoxantrone plus prednisone with a 24% relative reduction for men with metastatic CRPC and a significant survival benefit (*P* = 0.009) in the docetaxel arm [11, 30]. Docetaxel was also effective in pain reduction (35% versus 22%) (*P* = 0.001) [11, 30]. In SWOG 9916 trial, docetaxel plus estramustine was compared with mitoxantrone plus prednisone and the docetaxel regimen also conferred a significant survival benefit (HR for death 0.80; 95%  CI = 0.67–0.97) and increased median survival (17.5 versus 15.6 months) (*P* = 0.02) over the mitoxantrone arm [32, 57].

Several docetaxel combinations have been evaluated in phase 2 studies for CRPC, including associations with tyrosine kinase inhibitors, antiangiogenesis agents, and immunologic agents [32, 58]. Phase III trials, combining docetaxel with other chemotherapy agents, did not demonstrate superiority to docetaxel plus prednisone [31].

Epothilones, namely, ixabepilone and patupilone, have shown significant activity in men with CRPC [31, 59–62]. These molecules were evaluated in second-line chemotherapy in two phase II trials after progression with prior taxane [63, 64]. Phase III trials with ixabepilone are in development and a phase II trial of patupilone is currently underway [31].

Eribulin mesylate (E7389) is a synthetic analog of the marine macrolide halichondrin B, which acts as a novel microtubule modulator with a distinct mechanism of action (different from taxanes) [31, 63]. An open-label, multicenter, single-arm, phase II study was conducted in patients with CRPC stratified by prior taxane therapy [31, 65]. Primary efficacy endpoint was PSA response rate defined as two consecutive ≥50% decreases in PSA levels from baseline. The secondary endpoints were duration of PSA response rate and objective response rate by RECIST criteria. One hundred and eight patients were available for analyses. Of these 50 were taxane pretreated. Eribulin showed activity in patients with metastatic CRPR, especially in those with taxane naïve disease. Side effects, mainly hematological toxicity (grade 3 and 4 leucopenia and neutropenia), fatigue, and peripheral neuropathy were manageable [66].

Satraplatin (JM-216) is an oral third-generation platinum compound evaluated in the SPARC trial in combination with prednisone in second-line therapy after docetaxel [3, 18]. In this trial, satraplatin plus prednisone resulted in significant improvement in PFS (11.1 weeks versus 9.7 weeks) (*P* < 0.001) but there were no improvement in median overall survival compared with prednisone alone (61.3 weeks versus 61.4 weeks) (*P* = 0.80).

Cabazitaxel, a novel tubulin-binding taxane, is the first chemotherapy shown to improve survival in patients with docetaxel-refractory metastatic castration resistant prostatic cancer. In the TROPIC trial, a randomized phase III study compared cabazitaxel plus prednisone versus mitoxantrone plus prednisolone, in patients with docetaxel-refractory prostate cancer. The cabazitaxel arm showed an improvement in median PFS (2.8 months versus 1.4 months) (*P* < 0.0001), median OS (15.1 months versus 12.7 months), and lower risk of death (hazard ratio 0.70) (*P* < 0.0001) [8, 14, 66].

### 3.5. Vaccines-Based Immunotherapy

Sipuleucel-T (Provenge, APC8015) is an autologous dendritic cell vaccine, consisting of autologous peripheral blood mononuclear cells (PBMCs), including antigen-presenting cells (APCs), that have been activated *ex vivo* with a recombinant fusion protein (PA2024) composed of prostatic acid phosphatase (PAP) linked to granulocyte-macrophage colony-stimulating factor (GM-CSF) [15]. In the first two randomized trials, sipuleucel-T, the primary endpoint was not accomplished since these studies did not show a significant effect on the time to disease progression comparing with placebo. Despite this, the hazard ratios were in favor of sipuleucel-T [67, 68]. The subsequent IMPACT trial, a phase III, randomized trial, in patients with asymptomatic or minimally symptomatic metastatic CRPC, designed overall survival as the primary end point. This study resulted in a 4.1-month improvement in median overall survival and an improvement in the rate of 3-year survival (31% versus 23%) in sipuleucel-T arm, with limited toxicity. However, no significant effect on the time to objective disease progression was observed [15].

GVAX (CGI940/CG8711) is a cellular vaccine composed of two allogeneic prostate cancer cell lines (LNCaP and PC-3) that is genetically modified to secrete GM-CSF [17]. This vaccine showed clinical benefit with limited toxicity in phase I and II trials [18, 69, 70]. However, the two phase III trials (VITAL-1 and VITAL-2) evaluated GVAX against docetaxel plus prednisone in naïve CRPC and both were closed prematurely [18, 31]. The VITAL-1 study was closed when the unplanned futility analysis revealed a <30% chance of meeting its predefined primary endpoint of OS improvement and the VITAL-2 terminated when an interim analysis revealed more deaths in the GVAX arm than in the control [18, 31, 33, 71].

PROSTVAC-VF is a cancer vaccine consisting of a recombinant vaccinia vector as a priming immunization with subsequent multiple booster vaccinations, using a recombinant fowlpox vector. This agent presented in the context of 3 costimulatory molecules (ICAM-1, BLA-7, and LFA-3) which, when taken together, demonstrate an increase in strength of the target immunologic response [31]. This vaccine was evaluated in phase I and II trials. The phase I trial showed PSA stabilization in 40% of patients and limited toxicity and, in the phase II study, patients in the PROSTVAC-VF arm achieved an 8.5-month improvement in median OS (25.1 months versus 16.6 months) and a 44% reduction in the death rate (Hazard ratio 0.56, *P* = 0.0061) [72, 73]. Phase III trial are being planned and other vaccines are under current development [74].

## 4. Other Targets

The Endothelins (ETs) constitute a family of three 21-amino-acid peptides (ET-1, ET-2, and ET-3) that are synthesized as propeptides and are transformed to their active forms by sequential endopeptidase and ET-converting enzyme-mediated cleavage [79]. ETs are regulators of cell proliferation, vasomotor tone, and angiogenesis [31]. The ETs bind to two receptors, endothelin-A (ET-A) and endothelin-B (ET-B), and play an important role in angiogenesis, proliferation, escape from apoptosis, invasion, tumor growth, new bone formation, and bone metastasis [31, 74]. ET and their receptors have emerged as a potential targets in CRPC [74, 79]. Efficacy and safety of ET-A receptor blockade—atrasentan (ABT-627)—have been evaluated in a double-blind, randomized, placebo-controlled, phase II trial [79]. Two hundred and eighty-eight asymptomatic patients were randomized to one of three study groups: placebo, 2.5 mg atrasentan, 10 mg atrasentan. Primary endpoint was time to progression. Secondary end points were time to PSA progression, bone scan changes, and changes in bone and tumor markers. Target therapy with atrasentan was well tolerated and results showed a potential to delay progression of CRPC.

Based on these results other phase III studies also evaluated atrasentan. In one of these studies [75] atrasentan did not reduce the risk of disease progression relative to placebo. However exploratory analyses showed that alkaline phosphatase and PSA levels were significantly lower in the treatment arm [31]. Another phase III study (SWOG S0421) tested atrasentan combined with docetaxel/prednisone in metastatic CRPC as a first-line therapy [80]. SWOG trial S0421 closed earlier based on interim finding that atrasentan added to docetaxel and prednisone did not confer additional survival benefit to patients with hormone-refractory prostate cancer.

The Data and Safety Monitoring Committee has determined that patients in phase III S0421 receiving atrasentan in addition to a standard chemotherapy regimen for advanced prostate cancer did not have longer survival or longer progression-free survival. Zibotentan (ZD 4054) is another ET-A receptor antagonist, which showed evidence of activity in a randomized phase II trial in men with castrate-resistant prostate cancer and bone metastases [81]. Following these results two phase III trials [82, 83] were conducted. ENTHUSE M0 was discontinued following the results of an early efficacy review by the Independent Data Monitoring Committee. The company has concluded that zibotentan was unlikely to meet its primary efficacy endpoints progression free survival and overall survival. Results from ENTHUSE M1C are still awaited.

Angiogenesis inhibitors such as thalidomide and bevacizumab alone or in combination with docetaxel were studied in phase II trials with promising results. Thalidomide plus docetaxel versus docetaxel monotherapy, in a phase II trial in patients with metastatic CRPC, showed a ≥50% PSA decrease (53% versus 37%) (*P* = 0.32) and improvement in median overall survival (28.9 months versus 14.7 months) (*P* = 0.11) for patients in the thalidomide group [18, 84].

Bevacizumab, a recombinant humanized monoclonal antibody anti-VEGF, was studied in a phase II, in patients with docetaxel-refractory CRPC. Bevacizumab plus docetaxel resulted in ≥50% PSA reduction in 55% of patients, 37.5% partial responses, and a median overall survival of 9 months [85]. Bevacizumab, docetaxel and estramustine resulted in >50% PSA reduction in 75% patients partial response in 59% of patients and median overall survival of 24 months [86]. However, phase III, CALGB 90401 trial did not show improvement in OS (22.6 months versus 21.5) with the addition of bevacizumab to docetaxel [18, 76].

The combination of docetaxel, thalidomide, bevacizumab, and prednisolone was also evaluated in a phase II trial with a ≥50% PSA reduction in 89.6% of patients. The median time to progression was 18.3 months and the median overall survival was 28.2 months [87]. More studies are needed before prescribing angiogenesis inhibitors outside clinical trials.

Src inhibitors, such as dasatinib, are being studied for prostate cancer because Src signaling is involved in androgen-induced proliferation. In a phase II trial in chemotherapy-naïve patients with metastatic CRPC, dasatinib (100 mg orally twice daily) showed lack of progression in 43% of patients at week 12 and in 19% in patients at week 24. It also revealed a decrease in the markers of bone metabolism (N-telopeptide and bone alkaline phosphatase) [31]. A randomized phase III trial with dasatinib plus docetaxel is ongoing [88].

Blockade of the T-cell inhibitory receptor CTL-associated antigen-4 (CTLA-4) augments and prolongs T-cell responses and is a strategy to elicit antitumor immunity [89]. Ipilimumab, an anti-CTLA-4 antibody, was tested in order to potentiate endogenous antitumor immunity to prostate cancer through combination immunotherapy with CTLA-4 blockade and GM-CSF [90]. The results showed that this combination immunotherapy can induce the expansion not only of activated effector CD8 T cells *in vivo* but also of T cells that are specific for known tumor-associated antigens from endogenous immune repertoire.

In a pilot trial of CTLA-4 blockade with ipilimumab patients with CRPC were given a single dose of 3 mg/kg [89]. Results showed that this approach was safe and did not result in significant clinical autoimmunity. PSA modulating effects presented need further investigation in order to be fully understood.

Two phase III trials are now recruiting patients in order to compare ipilimumab with placebo [88]. One trial [91] will evaluate this approach in patients with metastatic disease, with at least one bone metastasis, prior treatment with docetaxel, and castrate levels of serum testosterone. The other trial [77] will include patients with metastatic castration-resistant prostate cancer who are asymptomatic or minimally symptomatic and who have not received prior chemotherapy or immunotherapy.

Tyrosine kinase inhibitors (TKIs) are important new class of target therapy that interfere with specific cell signaling pathways and thus allow target specific therapy for selected malignancies. Sorafenib and sunitinib have been tested in prostate cancer in phase I and II trials.

In the first stage of a phase II trial with sorafenib [92] 22 metastatic CRPC were enrolled. Most of the patients (59%) had received prior therapy with docetaxel or mitoxantrone. Sorafenib therapy failed to show >50% PSA reduction [18]. A second stage of the trial was conducted with 24 more patients [93]. Of the 24 patients, 21 had previous chemotherapy with docetaxel. All patients had bony metastases, either alone (in 11) or with soft-tissue disease (in 13). One patient had a partial response; 10 patients had stable disease (median duration 18 weeks, range 15–48). At a median potential followup of 27.2 months, the median progression-free survival was 3.7 months and the median overall survival was 18.0 months. For the whole trial of 46 patients the median survival was 18.3 months. The authors concluded that sorafenib has moderate activity as a second-line treatment for metastatic castration-resistant prostate cancer in this trial population [21, 94].

Another phase II study [77] included 57 chemotherapy naïve CRPC patients. Fifty-five patients were evaluable. Two of these patients had >50% PSA reduction and 15 patients had stable disease. Analysis of the results from a third phase II trial suggests that sorafenib therapy could affect PSA production or secretion regardless of its antitumor activity [21, 95].

A phase I/II trial of sunitinib in combination with docetaxel and prednisone showed a PSA response in 56% of patients, a median time to PSA progression of 42.1 weeks, and a partial response of measurable disease in 39% patients [96].

Sunitinib was also tested in CRPC naïve and docetaxel refractory patients in other phase II trials [94, 95]. A phase III trial comparing sunitinib plus prednisone versus prednisone alone, in patients with docetaxel refractory metastatic CRPC, is ongoing. Overall survival is the primary endpoint of this study [18].

Cabozantinib is an inhibitor of MET and VEGFR2 [70]. Both the MET and VEGF-type 2 receptor signaling pathways appear to play important roles in the function of osteoblasts and osteoclasts. MET signaling promotes tumor growth, invasion, and metastasis. Results from cabozantinib trial were presented at ASCO Meeting, 2011. The authors concluded that cabozantinib showed clinical activity regardless of prior docetaxel in metastatic CRPC patients, particularly in patients with bone disease, in addition to improvements in hemoglobin and tumor regression.

ARQ-197 is an oral, selective, nonadenosine triphosphate competitive c-MET inhibitor [97]. Results from this clinical trial showed that ARQ 197 safely inhibited intratumoral c-MET signaling. Further clinical evaluation focusing on combination approaches is ongoing. Based on the first reports promising developments are expected.

There are also other potential targets, such as IGF-1R signaling, vitamin D receptor, PTEN, and phosphoinositide 3-kinase signaling; those are quite promising and could lead us to new treatment options [3].

Table 1 summarizes the main studies and the therapeutic impact of new drugs in CRPC treatment.

## 5. Conclusions

Androgen-deprivation therapy is generally the initial treatment for men with advanced prostate cancer. Different approaches include orchiectomy, LHRH agonist, or a combination of an LHRH agonist plus an antiandrogen (complete androgen blockade). Although patients have high response rates to the initial hormone therapy, nearly all of them eventually develop progressive, metastatic castrate-resistant, disease. In these patients other approaches are needed.

We know now that many of these CRPC tumors remain androgen dependent or AR stimulation dependent. Therefore it is possible that these patients benefit from sequential hormonotherapy (e.g., abiraterone acetate) as well as other new chemotherapy agents or biological approaches.

Individual target therapy is not yet available at this time, but remains a goal.

Current knowledge about the resistance mechanisms in castration-resistant prostate cancer has lead to new experiments and has identified possible new therapeutic targets. Promising results have already been presented in a broader spectrum of options. However, the survival benefit of these drugs in CRPC is still modest and some of the previous therapeutic options are not yet safe outside clinical trials. Therefore, well design and with potential clinical impact phase III trials are warranted, to coroborate the preliminary results and to answer unmet needs in CRPC.

## Figures and Tables

**Table 1:** Summary of the therapeutic impact of new drugs in CRPC treatment.

	Reference, study phase, and patient number	Efficacy results PFS*, OS^+^	Comparator	PSA levels/PSA RR^#^
Docetaxel	Tannock et al. [13] Phase III; 1006 pts	Prolongation of median survival, decrease in serum PSA level, predefined reductions in pain and improvements of quality of life	Mitoxantrone	Reduced

Cabazitaxel	Bono et al. [65] Phase III; 755 pts	Improved OS (95% CI: 0.59–0.83, *P* < 0.0001) and median PFS (HR: 0*·*74, 0.64–0.86, *P* < 0.0001)	Mitoxantrone	Reduced

Abiraterone acetate	De Bono et al. [26] Phase III; 1195 pts	Prolongation of OS, time to PSA progression (10.2 versus 6.6 months; *P* < 0.001), progression-free survival (5.6 months versus 3.6 months; *P* < 0.001), and PSA response rate (29% versus 6%, *P* < 0.001)	Placebo	Reduced (>29%)

Bicalutamide	Kucuk et al. [22] Phase II; 52 pts	Decreases pain and improves symptom status	Baseline after 1st line	Reduced (≥50% in 20% pts)
	Lodde et al. [23] Prospective trial; 38 pts	PSA response rate	Baseline after antiandrogen manipulation	Reduced (44.7% pts)

DES	Smith et al. [25] Phase II; 21 pts	PSA response rate	Baseline after 1st line hormonal therapy	Reduced (43% RR)

Sipuleucel-T	Kantoff et al. [15] Phase III, 512 pts	Relative reduction of 22% in the risk of death as compared with the placebo group (hazard ratio, 0.78; 95% confidence interval (CI), 0.61 to 0.98; *P* = 0.03)	Placebo	Reduced (≥50% in 2.6%)

MDV3100	Scher et al. [34] Phase I-II Phase III results not available	NA	NA	NA

Patupilone	Beardsley et al. [63] Phase II, 83 pts	Prolongation of PFS, PSA declines, and pain response (decline)	Docetaxel	Reduced (≥50% in 46%)

Eribulin	Bono et al. [65] Phase II, 108 pts	PSA response rate	Baseline (stratified by prior taxane exposure)	Reduced
PROSTVAC-VF	Kantoff et al. [72] Phase II; 125 pts	44% reduction in the death rate and an 8.5-month improvement in median OS	Control empty vectors plus saline injections	NA

Atrasentan (plus Docetaxel)	Carducci et al. [75] Phase III; 809 pts	Alkaline phosphatase and PSA levels were significantly lower in the treatment arm	Docetaxel and placebo	Reduced

Bevacizumab (plus Docetaxel)	Kelly et al. [76] Phase III; 1050 pts	Improvement in PFS, measurable disease response, and posttherapy PSA decline	Placebo	Reduced (≥50% in 69.5%)

Ipilimumab	NCT01057810 [77] Phase III ongoing	NA	Placebo	NA

Sunitinib	Sonpavde et al. [78] Phase II; 36 pts	PFS, PSA decline, pain control	NA	Reduced (≥50% in 12,1%)

*PFS: progression free survival; ^+^OS: overall survival; ^#^RR: response rate; pts: patients; NA: not available.
